# On the Investigation of Surface Integrity of Ti6Al4V ELI Using Si-Mixed Electric Discharge Machining

**DOI:** 10.3390/ma13071549

**Published:** 2020-03-27

**Authors:** Muhammad Umar Farooq, Mohammad Pervez Mughal, Naveed Ahmed, Nadeem Ahmad Mufti, Abdulrahman M. Al-Ahmari, Yong He

**Affiliations:** 1 Department of Industrial and Manufacturing Engineering, University of Engineering and Technology, Lahore 39161, Pakistan; umarmuf0@gmail.com (M.U.F.);; 2 Industrial Engineering Department, College of Engineering and Architecture, Al-Yamamah University, Riyadh 11512, Saudi Arabia; 3 Department of Industrial Engineering, College of Engineering, King Saud University, Riyadh 11421, Saudi Arabia; alahmari@ksu.edu.sa; 4 Key Laboratory of 3D Printing Process and Equipment of Zhejiang Province, School of Mechanical Engineering, Zhejiang University, Hangzhou 310027, China; yongqin@zju.edu.cn

**Keywords:** bio-active surface, osseointegration, electric discharge machining, surface integrity, surface roughness, recast layer thickness, nano-porosity, biomedical implants, Ti alloy

## Abstract

Surface modification is given vital importance in the biomedical industry to cope with surface tissue growth problems. Conventionally, basic surface treatment methods are used which include physical and chemical deposition. The major drawbacks associated with these methods are excessive cost and poor adhesion of coating with implant material. To generate a bioactive surface on an implant, electric discharge machining (EDM) is a promising and emerging technology which simultaneously serves as machining and surface modification technique. Besides the surface topology, implant material plays a very important role in surgical applications. From various implant materials, titanium (Ti6Al4V ELI) alloy is the best choice for long-term hard body tissue replacement due to its superior engineering, excellent biocompatibility and antibacterial properties. In this research, EDM’s surface characteristics are explored using Si powder mixed in dielectric on Ti6Al4V ELI. The effect of powder concentration (5 g/L, 10 g/L and 20 g/L) along with pulse current and pulse on time is investigated on micro and nanoscale surface topography. Optimized process parameters having a 5 g/L powder concentration result in 2.76 μm surface roughness and 13.80 μm recast layer thickness. Furthermore, a nano-structured (50–200 nm) biocompatible surface is fabricated on the surface for better cell attachment and growth. A highly favourable carbon enriched surface is confirmed through EDS which increases adhesion and proliferation of human osteoblasts.

## 1. Introduction

Titanium and its alloys are being used in automotive, aeronautical, biomedical and a number of other industries due to their exceptional properties. Titanium alloy Ti6Al4V ELI (grade 23) is solely developed by in view of biomedical industry applications [1,2]. It contains low levels of interstitials that have improved mechanical and thermal properties than grade 5. These properties include fracture and corrosion resistance, wear and cryogenic properties [3,4]. A number of machining methods are employed to fabricate desired shapes. But, due to its hard to cut limitation, conventional machining processes are not preferable for special purpose applications.

Non-conventional machining techniques electric discharge machining (EDM), electron beam machining (EBM), and laser beam machining (LBM) among many others are being used to machine hard-to-cut materials [5]. In addition to machining the profiles, biomedical applications in dental and orthopaedics require specific surface modifications to enhance inherent (biocompatibility) properties. These processes are subject to continuing challenges to devise the most efficient machine settings which generate a good surface of the artificial bone (implant), which ultimately lead to enhanced properties [6]. Though electron beam melting is a widely used additive manufacturing process for fabricating different complex shapes [7] in the biomedical industry, the challenge remains in dealing with the intrinsic changing surface roughness in between layers due to layer by layer deposition offering potential places for fatigue cracks to initiate [8,9]. Therefore, EDM is considered to be likely choice for machining as well as surface modification process due to having the potential to improve the mechanical properties, corrosion and fatigue resistance, improved surface hardness, fabricating the most favourable surface on bio-implant due to generation of carbon and oxygen-enriched surface [5,10,11]. Electric discharge machining uses a series of high-frequency sparks that erode material by melting, vaporization, simultaneous solidification, again melting and thus on [12]. 

Some of the advances are done recently in the EDM process in which assistive technologies and concepts are used. These include EDM with powder and/or surfactant mixed dielectric [13], ultrasonic-assisted machining [14], EDM fast hole drilling [15], magnetic stirring [16] and rotary machining etc. The mixing of powder, surfactant and different type of gasses in the dielectric to enhance properties of dielectric or to deposit a specific required layer on the surface in EDM is called additive mixed electric discharge machining. During machining, mechanical properties of workpiece material interfere least, making the process preferable for difficult to cut materials. Though there are some challenges regarding the process e.g., controlling material migration and deposition on the surface [6], recast layer thickness, surface roughness, material removal rate, dimensional accuracy and tool wear rate etc. due to complex nature of the process. 

Recently, the attention of researchers has been drawn towards the fabrication of a biomedically active surface using additive mixed electric discharge machining. It also significantly improves corrosion and wear resistance, fatigue life and biomedical properties of artificial human body components (implant). Powder-mixed EDM is utilized to deposit a nano-porous and biocompatible layer on the machined surface of the implant [6,17]. According to the biocompatibility and mechanical properties, different bioimplants are used for the short term, long term or even permanent if not removed surgically. Machining process has a significant impact on life and quality of implants, especially on permanent bioimplants. Electric discharge machining results into a biocompatible surface that affects osseointegration of implant with body tissues, bones and environment of the body. In comparison to conventional surface modification techniques like PVD, CVD, sol-gel, anodization and thus on, EDM shows better potential for surface modification [18,19]. The benefits associated with the EDM regarding the machining of bioimplants are:

- No surface preparation required before EDM;
- Ability to generate a layer of oxides and carbides of controlled thickness on the surface which enhance biocompatibility and generates hydrophilic surface;
- Ability to produce nano-porous surface;
- Fabricates the most favourable surface for tissue growth;
- Improves wear and corrosion resistance;
- Improves surface hardness of the implant.

Different type of metals Ti and its alloys, Ni and its alloys, Steel and Magnesium (Mg) etc. are being used for bio-implant manufacturing. For the orthopaedic and dental applications, Ti and its alloys are used extensively. In particular, 50% of the overall consumption of pure Ti is of Ti6Al4V. During the EDM of Ti and its alloys, the effect of process parameters like pulse on time (Ton) and pulse current (Ip) are found to the most prominent on surface integrity characteristics [20]. 

Recently, Jahan et al. [21] studied material migration and surface composition of Ti6Al4V (grade 5) using tungsten carbide electrode. Besides electrode material transfer, migration of carbon and oxygen was confirmed due to decomposition of hydrocarbons in the dielectric. A similar study was carried out by Chundru [22] on Ti6AL4V with TiC/Cu powdered electrode. Lee et al. [23] used tungsten carbide powder in kerosene to carry out surface modification along with Ton and Ip machine steel. The study resulted in 57.98% and 129.17% improvements in the surface finish and microhardness of powder mixed electric discharge machining (PMEDM), respectively, as compared to EDM. Another researcher carried out similar study with titanium powder mixed dielectric and using pulse on:off time, current, polarity, and copper electrode as variables to evaluate surface characteristics (topography, recast layer thickness) [24]. Opoz et al. [25] evaluated surface modification of Ti6Al4V using Hydroxyapatite powder for medical applications. Powder concentration was found to be one of the influential factors to obtain surfaces which enhance biocompatibility and cellular activity. Bui et al. [26] studied EDM for antibacterial coatings on the titanium implant surface. The deposit of powder increased with the increase in powder concentration and surface roughness decreased up to a certain level. Das et al. [27] used bio-dielectrics to machine Ti6Al4V with Cu electrode and found that surface roughness (SR) is decreased by bio-dielectrics as compared to kerosene. Tong et al. [28] worked on rough and finish machining to generate 3D cavities. Liang et al. [29] compensated tool wear rate to achieve accuracy. Prakash et al. [30] evaluated Si mixed EDMed surface modification of Ti-β alloy. Powder concentration (4 g/L) was found to be the most optimum concentration which resulted in least surface roughness. Recast layer thickness (RLT) was increased with the increase in powder concentration. Kou et al. [31] used moving arcs in EDM to machine Ti6Al4V. The study revealed that workpiece could be machined with higher efficiency due to continuous machining. Kumar et al. [32] machined titanium for biomedical application and found Ton and voltage most significant parameters on surface roughness. Ying et al. [33] revealed that RLT increased with the increase in Ip while machining with diamond abrasives. 

It is evident from the literature that most of the researchers have evaluated the machining efficiency of different materials. The experimental researches are at initial stages regarding the surface modification through EDM in context of biocompatibility. EDM has been used for coating [26,34,35], particle migration [36,37], layer quality improvement [30,38,39], and nano-surface treatment [30,40,41,42] for biomedical implants surface treatment of titanium alloys [43]. 

However, machining of Ti6Al4V ELI grade 23, also known as Titanium medical-grade, through PMEDM is hardly reported and there are no comprehensive studies available that shows a significant exploration of PMEDM parameters on surface and machining characteristics of Ti6Al4V ELI grade 23 such as surface topography, surface cracks, porosity, SR, RLT and elemental composition. Therefore, there is an urgent need of study to explore powder concentration along with pulse on:off time and pulse current which significantly impacts on surface modification. So, in the present research work, silicon particle mixed dielectric fluid in EDM with different concentrations is evaluated to enhance biocompatibility by producing favourable surface characteristics. 

## 2. Materials and Methods

In the present study, a medical-grade alloy of titanium (Ti6Al4V ELI grade 23) is used as workpiece and its chemical composition has been confirmed by optical image spectroscopy as stated in Table 1. Since EDM is thermal erosion process in which series of electric discharges perform melting and vaporization of material on interaction area making the process dependent on electrical and thermal properties of the material. The salient properties of the workpiece which affect machinability and biocompatibility are mentioned in Table 2. 

A sheet of 300 × 100 × 2.5 mm^3^ (length × width × thickness) was used to perform experimentation with Cu electrode. Cu electrode offers a better surface finish. Moreover, Cu exhibits special functions to biomedical alloys such as enhancing mechanical properties through solution strengthening, improves biocorrosion resistance and provides antibacterial properties [44]. Si powder was used in the dielectric to enhance machining properties. The characteristics of Si powder are described in Table 3. 

**Figure 1:**
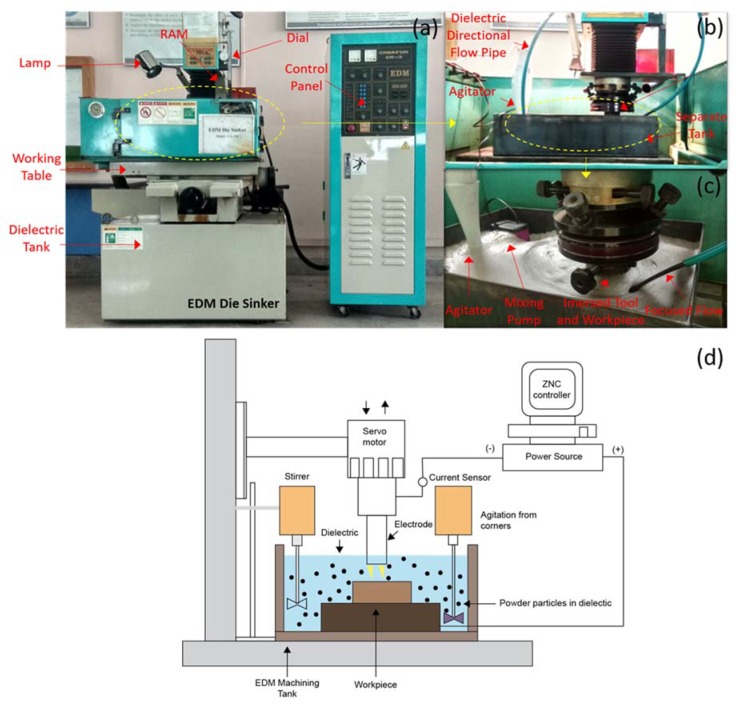
Experimental setup and schematic: (**a**) EDM setup as a whole, (**b**) closeup of machiing head, (**c**) closeup of mixing tank, and (**d**) schematic of powder mixed electric discharge machining.

**Figure 2:**
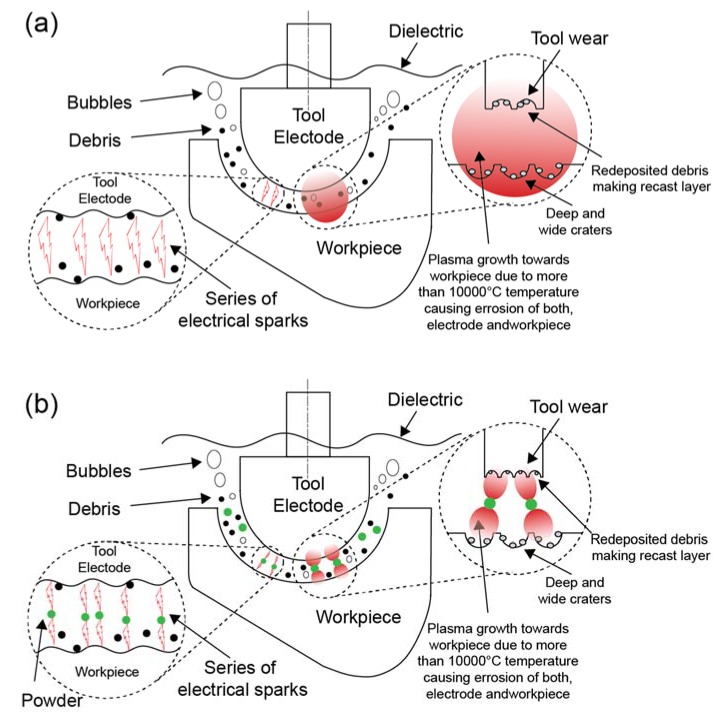
Schematic of process (**a**) EDM; (**b**) PMEDM.

An EDM die sinker (Model: Rj-230 manufactured by Creator, Taiwan) was utilized for experimentation with a separate setup to ensure proper mixing of powder. The setup and schematic of the process are shown in Figure 1a–d respectively. Figure 2 shows the plasma generation during EDM and PMEDM. The gap is increased during PMEDM and which improves the machined surface. 

**Figure 3:**
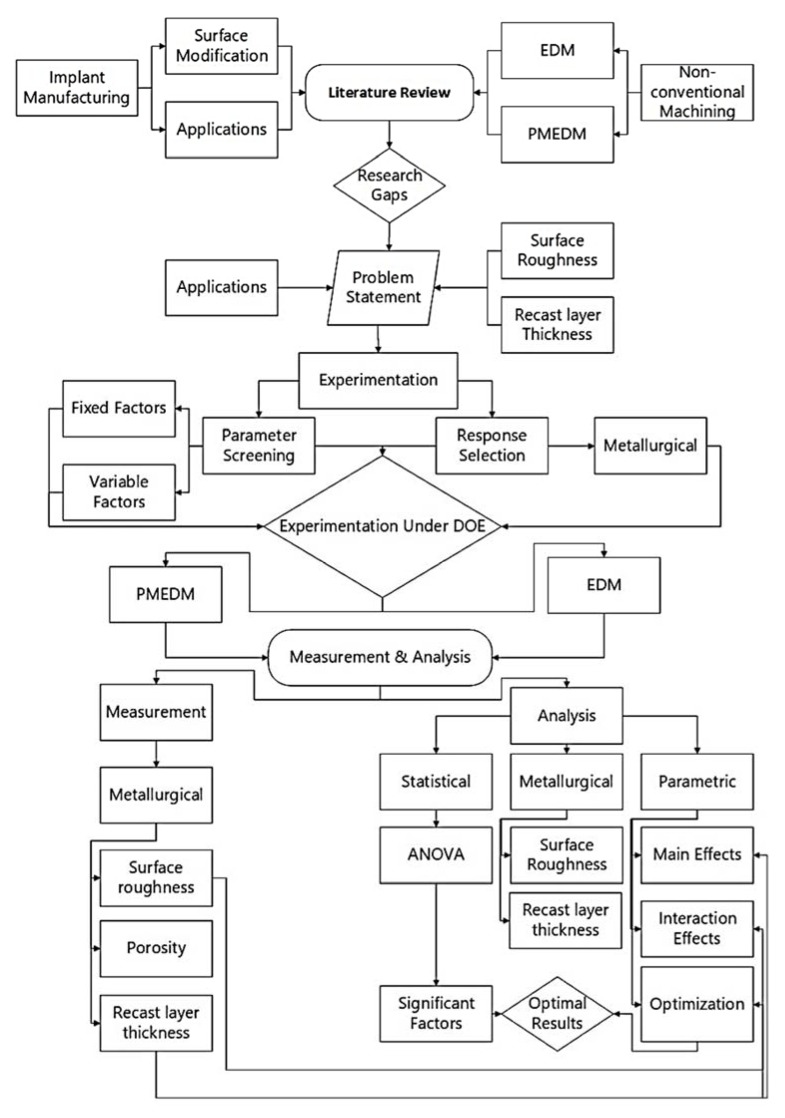
The overall methodology of the research project.

In trail runs, nine experiments were conducted with the positive and negative polarity of electrode. The evaluation criterion of initial trial experimentation included complete cut impression, lesser mild burns and lower surface roughness to be achieved in 15 minutes machining time. After that negative polarity of tool exhibited good results which was selected for mature experimentation. Furthermore, different values of pulse on:off and pulse current were also tested to generate the design of experiments. The parameters other than control parameters were kept constant. Full factorial design of the experiment is utilized with 15 mm copper electrode to carry out the study. In total 27 experiments with different concentrations of powder and 9 experiments with no powder in dielectric were conducted. Control and constant parameter values are mentioned in Table 4 and Table 5, respectively. The overall methodology to conduct the research is displayed in Figure 3. 

The surface roughness of machined samples was measured by surface texture meter (Surtronic 128 manufactured by Talor Hobson, UK) in three values Ra, Rz and Rt. Since Ra is the most widely accepted surface roughness value in the industry. Therefore, SR values in the study is in the form of Ra and an average of five consecutive values with 4 mm cutoff length. The topological analysis was examined on EDS equipped Quanta 450 Field Emission Gun (FEG) Scanning Electron Microscopy. The compositional analysis and re-solidified layer were identified and examined on INSPECT S50 Scanning Electron Microscopy. 

Machined samples were cross-sectioned by CNC Wire EDM (Model: CHMER G43S) and hot moulded then grinded by 200–2000 mesh size paper which were further polished on 0.5–1 μm diamond paste. The thickness of layers i.e., recast layer, heat affected zone (HAZ) was observed by taking an average of consecutive twenty readings by ImageJ software. Recast layer thickness was measured with the assistance of ImageJ software. Furthermore, results were analyzed by Minitab 19 software.

## 3. Results and Discussion

The present study investigates surface quality along with machining characteristics to modify the surface without compromising on machining efficiency. Surface characteristics (micro and nanoscale) which include SR, RLT, surface crack, porosity, and surface chemistry are critical for enhancing biocompatibility, adhesion, and osseointegration [18]. 

**Figure 4:**
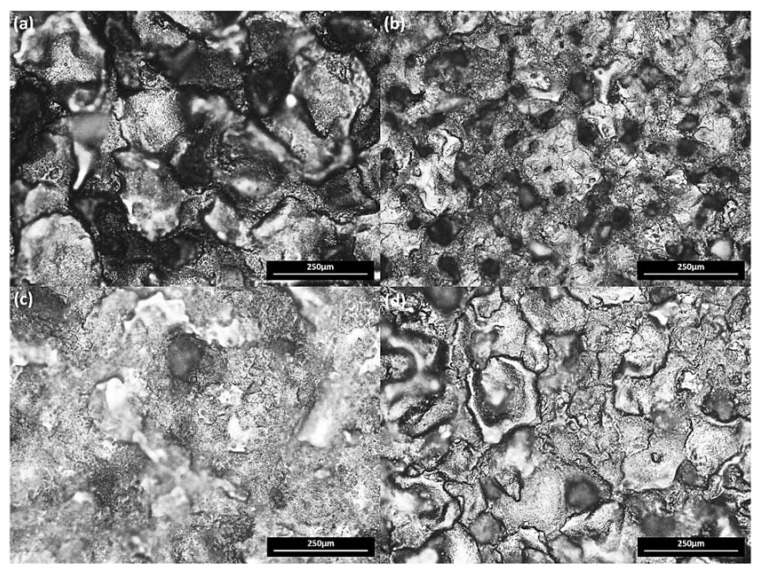
Surface Topography: Micrographs of Ti6Al4V ELI Grade 23 machined surface at 9 A pulse current on 200× magnification (**a**) EDM (**b**) PMEDM at 5 g/L (**c**) PMEDM at 10 g/L (**d**) PMEDM at 20 g/L.

Surface characteristics are directly proportional to discharge energy produced during the process. Figure 4 shows the micrographs of Ti-alloy surface at different conditions on 200× magnification. Figure 4a shows the machined surface of the workpiece using 0 g/L concentration of the powder. The shreds of evidence of redeposited molten metal, macroscale craters, micro-cracks, and pockmarks have been identified. The poor surface quality evidence is due to high discharge energy generation which proliferates and penetrates deeply into the workpiece leads to deeper and broader craters. High crack density and sharp-edged pits/craters of micron size (150–250 μm) were fabricated with a rough surface and poor quality. To prevent the poor surface quality, Si powder was added in the dielectric to enhance machining as well as surface characteristics as shown in Figure 2. Machined surface with 5 g/L powder concentration in Figure 4b shows less deep and small size craters due to the balanced distribution of discharges. The ridges of redeposited melted metal and overall craters become flat and interconnected with reduced density and size (20–50 μm). Similarly, interconnected and low height ridges have been observed in Figure 4c. The crack density and size were increased when the concentration of Si powder was increased from 10 g/L to 20 g/L. This is due to the fact of not properly dispersing the high discharge energy as shown in Figure 4d. As compared to EDM, lesser deep craters are observed at 20 g/L. It can be concluded that the presence of Si powder in dielectric during machining of Ti6Al4V ELI grade 23 improves surface characteristics and decreases surface defects. This is due to an increased electrode-workpiece gap and balanced distribution of discharge energy. Eroded debris flush away easily and form low height ridges which ultimately improves surface quality. 

**Figure 5:**
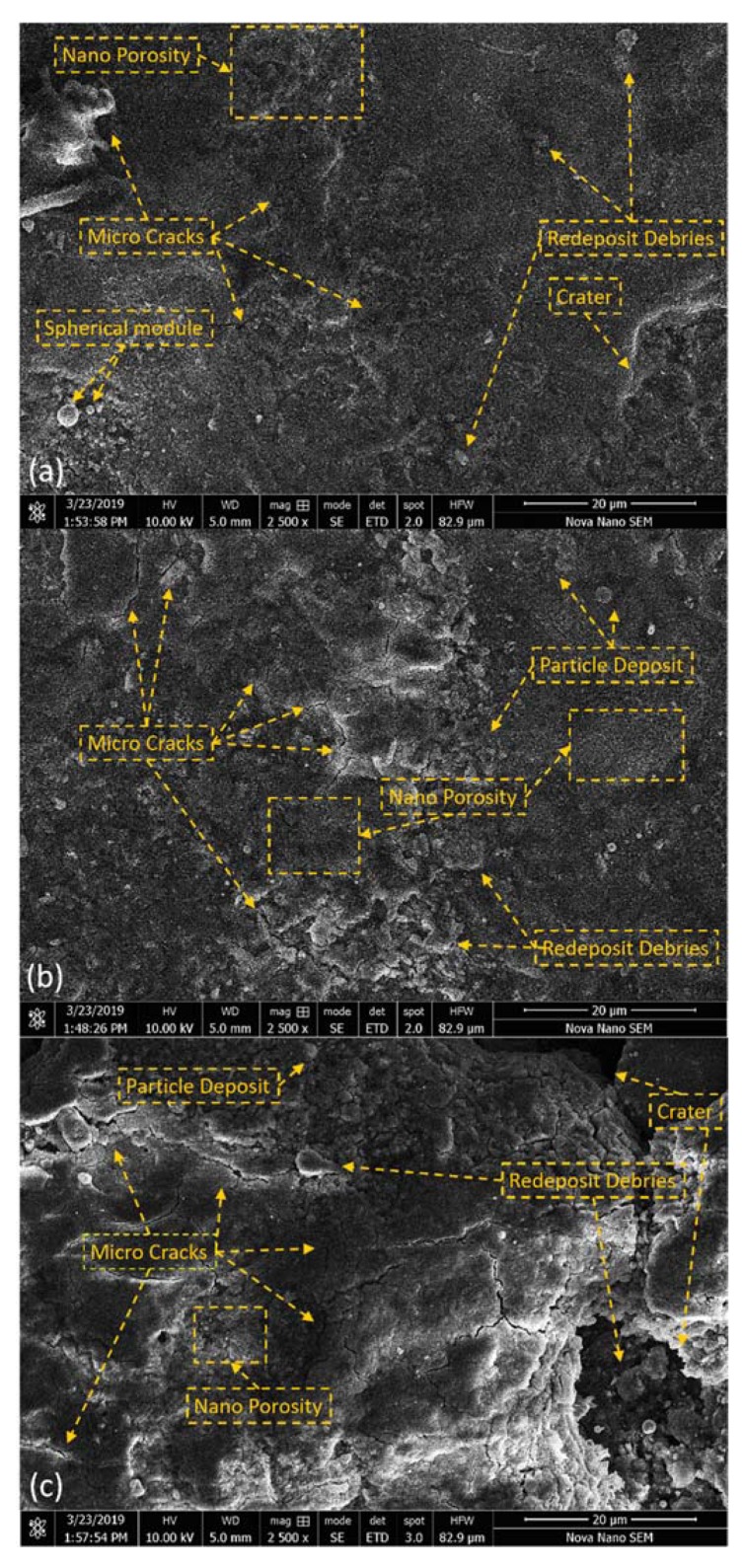
Surface Topography: SEM micrographs of modified surfaces using Si concentration (**a**) 5 g/L and 5 A Ip (**b**) 10 g/L and 7 A Ip (**c**) 20 g/L and 9 A Ip on 2500× magnification.

Figure 5 shows SEM micrographs of PM-EDMed surface on all the variable concentrations of powder. On 5 g/L Si concentration and 5 A Ip in Figure 5a, the observed surface has almost crack-free and smooth, no ridges and very low density of craters features which is comparatively of better-quality than EDMed surface in shown in Figure 4a. Low powder concentration (5 g/L) distributed discharge energy equally to produce smooth morphology. Furthermore, the cracks and surface irregularities have been increased when the concentration of powder is increased up to 10 g/L as shown in Figure 5b. By increasing powder concentration 10 g/L to 20 g/L, a further increase in crack density, ridges of molten metal, redeposited debris and pits/craters was observed which is the degradation of surface quality. Si powder is capable of distributing discharge energy and improving surface on low concentrations as compared to higher concentration as observed from Figure 5c. The surface characteristics are highly dependent on the electro-thermal energy being produced in the tool-workpiece gap. The influence of powder in breaking intense discharges is evident from Figure 5 to control surface irregularities caused by secondary discharges. Moreover, the Si traces in craters confirms the material migration phenomenon controlling machined surface quality. A similar study [36] on Ti6Al4V reported ~4.2 µm at 2 A current. However, the current study has improved the surface results by almost 50%.

**Figure 6:**
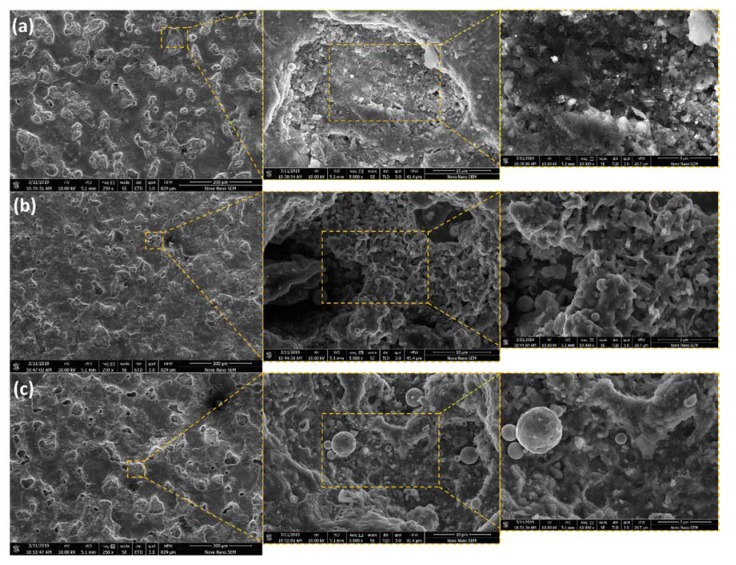
Surface morphology: Nano-porosity in PMEDMed modified surface of Ti6Al4V ELI Grade 23 on 5 A and Si powder concentrations (**a**) 5 g/L (**b**) 10 g/L (**c**) 20 g/L on 250×, 5000× and 10,000× magnifications.

Nano-porous features and nano-surface characteristic were observed on PMEDMed impressions. In the SEM micrographs of Ti6Al4V ELI grade 23, surface morphology can be observed on different concentrations of Si powder and Ip at 5 A, 7 A, 9 A and constant pulse on:off time 100:100 µSec as shown in Figure 6.

Figure 6a shows surface nano-porosity and micro-cracks on the machined surface at 250×, 5000× and 10,000× magnification on 5 g/L Si powder concentration. Porosity was measured on 25,000× and 50,000× magnification which appeared to be ~50–120 nm. Due to an increase in powder concentration from 5 g/L to 10 g/L, a significant decrease in nano-porosities was observed in Figure 6b. A further decline in surface nano-porosities density on the modified surface with 20 g/L Si concentration was observed as shown in Figure 6c. The characteristics of surface morphology on nano-scale have been significantly influenced by Si powder concentration. A similar trend [30] is observed in EDM of Ti β alloy. During machining with powder mixed in the dielectric, eroded debris are deposited again on the surface due to the quenching process which leads to path blockage for absorbed gases. These gases are released through nano-pores making nano-porosities and foam-like-shape surface. The decline in the density of nano-porosities is due to the increased gap between electrode and workpiece because of powder particles [46]. This increases the flexibility of flushing the eroded debris. As is evident from ANOVA (Table 6 and Table 7) of surface roughness and RLT, surface characteristics are majorly influenced through powder concentration. In addition to this, surface porosities are highly depended on powder concentration because it affects their density indirectly; when the concentration is increased, density of surface porosities is decreased. The reason being is, the powder helps to balance the discharges and directly influences in debris redeposit, removal and surface discharge energy distribution [42]. Hence, controlling the powder concentration will directly control surface porosities produced. It is reported in the literature that nano-surface characteristics facilitate artificial human body part surface in body environment for osseointegration or bone growth, growth of tissues by building biological interface on the surface for stability [43,47]. 

**Figure 7:**
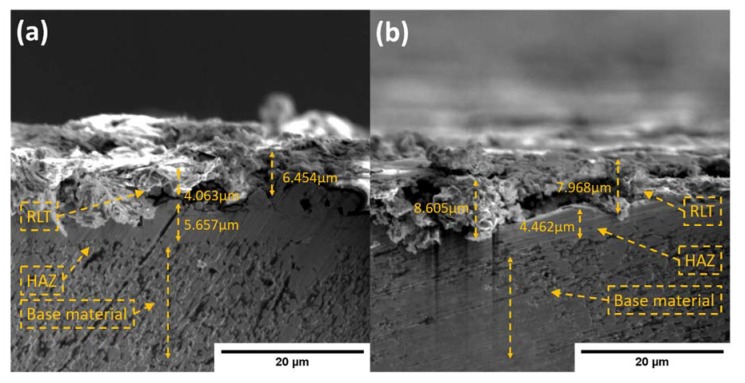
Recast layer thickness of machined surface at 5000× magnification with Si powder concentration (**a**) 5 g/L (**b**) 10 g/L.

It is reported in a number of findings that RLT has direct relation with powder concentration and pulse duration [30,36]. It mainly drives (surface hardness, corrosion resistance) mechanical characteristics of the machined surface. Figure 7 shows cross-section of machined sample SEM micrographs containing different surface layers produced during machining. The upper most layer having significant thickness is recast layer (RLT) which is produced due to solidification of eroded debris and powder particles. The layer below RLT is heat affected zone (HAZ) showing the traces of heat penetration in the material. This is sometimes considered as thermal stress and is unwanted in some machining applications through the process. In the bottom, the base material is present on which modification of surface is carried out. It is apparent that RLT is affected by powder concentration as can be seen dominating process parameter in Table 7, similar effect is observed during machining of different metals [30,48]. Increase in recast layer thickness was observed while increasing powder concentration because due to increased powder particles in electrode-workpiece gap, eroded debris entrapped and had no chance to flush out.

In our previous results, it is confirmed that Si-PMEDM has improved surface morphology on micro-scale by controlling surface characteristics (SR, craters, pits, ridges) and on the nanoscale by imparting porosity as well as controlling its density. These characteristics play a vital role in the osseointegration of implant and in increasing hydrophilicity [46,49]. 

**Figure 8:**
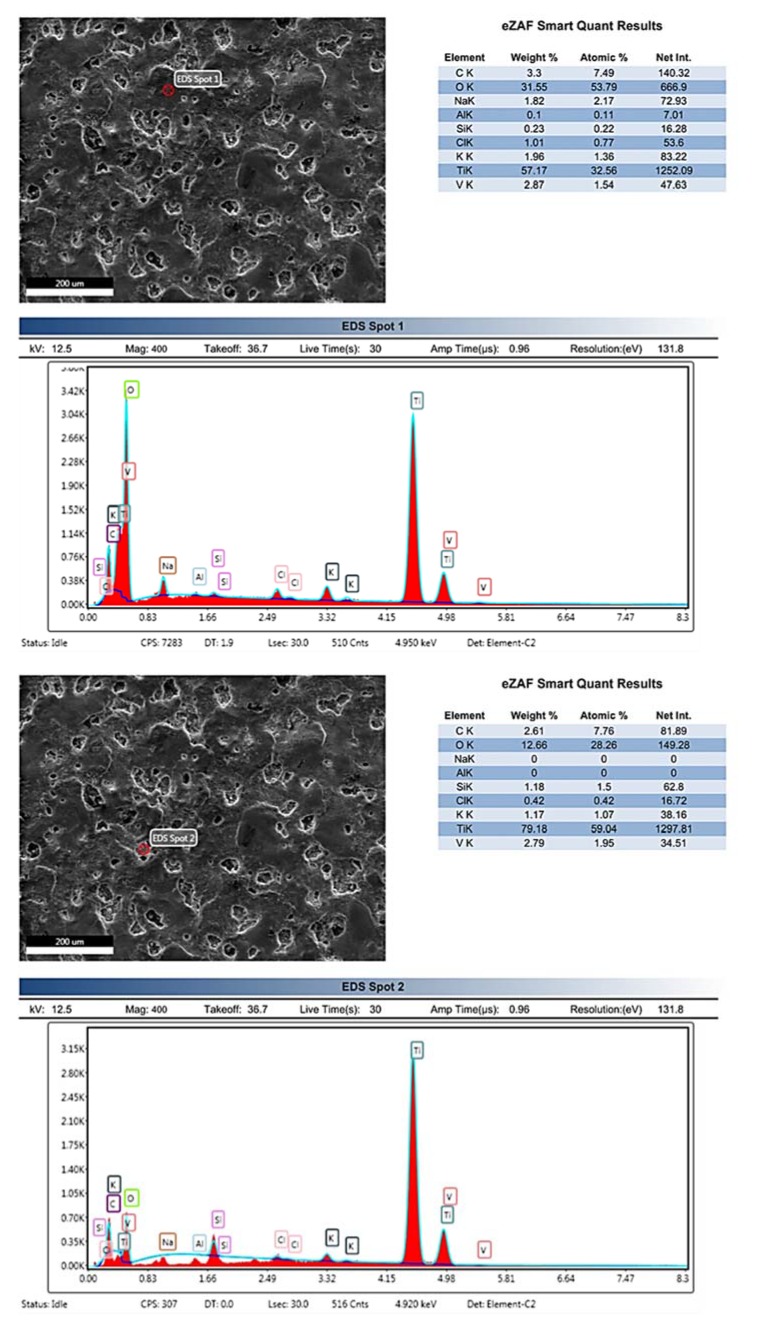
Energy Dispersive X-ray Spectroscopy: Si powder concentration 5 g/L, Ip 5 A, Ton 100 μSec (EDS Spot 1) Plain surface (EDS Spot 2) Crater.

In bioimplant manufacturing and surface modification, along with surface morphology, the surface composition is also very important to enhance biocompatibility and bone-implant anchorage. Different oxides and carbides (TiO_2_, SiO_2_, TiC etc.) are used previously in different studies to enhance biocompatibility [5,42]. In this study, EDS analysis is employed due to its capability to analyze the bulk concentration of elements present on the modified surface. The reason behind the depth of analysis is, tissue growth is integrated through micro (craters and roughness) and nano-scale topography (surface porosity) for better proliferation. Hence, elements present in bulk concentrations directly relates to affecting on osseointegration. Therefore, EDS analysis is done than XPS (which gives composition on the near-surface region only). Hence, EDS analysis confirms the deposition of Si particles from dielectric to the surface. Furthermore, oxygen (O) and carbon(C) exist on the surface due to decomposition of hydrocarbon oil (Kerosene oil used as dielectric during machining) in high spark energy. Presence of O and C confirms the formation of carbides and oxides in a higher proportion which may enhance surface properties along with deposition of Si. Figure 8(EDS Spot 1) shows the EDS spectrum on crater free surface which shows a higher proportion of O and C which is 31.55% and 3.3% respectively. In Figure 8(EDS Spot 2), the spectrum shows relatively higher migration of Si on the crater surface which is almost 1.18%. The traces of oxygen (O), carbon (C) and Si confirms particle migration from dielectric to surface. As a result, the surface chemistry and porous recast layer fabricated in this research work are considered as highly favorable for biomedical applications as previously reported in [6,36,41,43]. The decomposed elements formed oxides and carbides are highly likely to enhance surface hardness and bio activity. 

Table 6 and Table 7 show analysis of variance of surface roughness and recast layer thickness respectively. P-value of the factors shows the significance on the response. The confidence interval of 95% (α = 0.05) was decided to evaluate the significance. Any factor’s P-value less than 0.05 will be considered as a significant factor. In Table 6, powder concentration and its interaction with pulse current and pulse on time were significant factors with <0.0001, 0.001 and 0.018 respectively. Furthermore, the percent contribution of the factors on controlling the response is calculated by the ratio of Adj SS of each factor with the total sum of Adj SS. The percent contribution ratio of powder concentration was 61.82%. In Table 7, powder concentration was found to be significant (P-value = 0.30) on recast layer thickness response with the contribution 37.03%. All the remaining factors and interactions were insignificant on both responses. 

**Figure 9:**
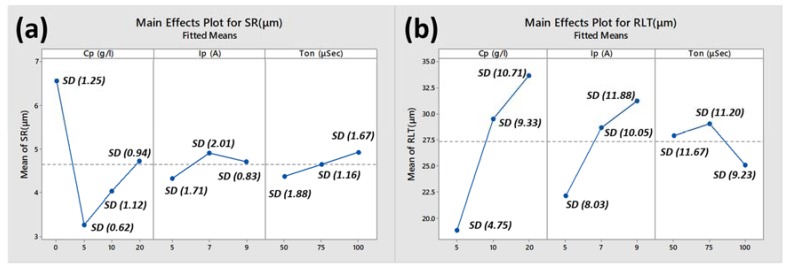
Main effect plots (**a**) surface roughness (**b**) recast layer thickness.

Figure 9 shows main effect plots of control factors on the responses along with standard deviation (in µm) of each variable level to show significance of data. Figure 9a shows combined effect of powder concentration, pulse current and pulse on time on surface roughness. Initially without Si mixed in dielectric (0 g/L concentration), surface roughness was more in the range of 6–7 μm as compared to 5 g/L concentration which resulted 3–3.5 μm as described earlier in Figure 4a,b respectively. This reduction in SR is due to the increased gap between electrode and workpiece, allowing debris to flush out effectively and the reduced amount of material in the form of micro debris were removed from the workpiece surface. As a result, micro-size craters were formed that improved the surface finish (decreased SR). At 10 g/L and 20 g/L, SR increased as compared to 5 g/L resulting 4–5 μm roughness as shown in Figure 4c,d. Higher concentrations of powder in this study could not distribute balanced discharges resulting in an increase. Unbalanced distribution of energy causes the creation of ridges and craters on the surface. Similarly, by the increase in pulse current and pulse on time, SR increased due to high spark energy developed causing immense melting and damaged surface as result in the larger and deep crater are formed which ultimately causes an increase in SR value.

RLT trends are shown in Figure 9b. RLT increased (average 10 μm to 29 μm to 34 μm) with the increase in powder concentration (5 g/L to 10 g/L to 20 g/L) due to insufficient flushing of eroded debris from the melted pool. Due to increase in pulse current (5 A to 7 A to 9 A), high discharge energy penetrates deeper which ultimately cause molten metal to re-solidify on the surface (average ~22.5 μm to 26.5 μm to 31 μm). With the increase in pulse on time, the thickness of the solidified layer increased initially and then decreased when pulse on-time became equal to pulse off-time. Eroded debris could flush out easily on higher pulse duration though there was higher discharge energy.

**Figure 10:**
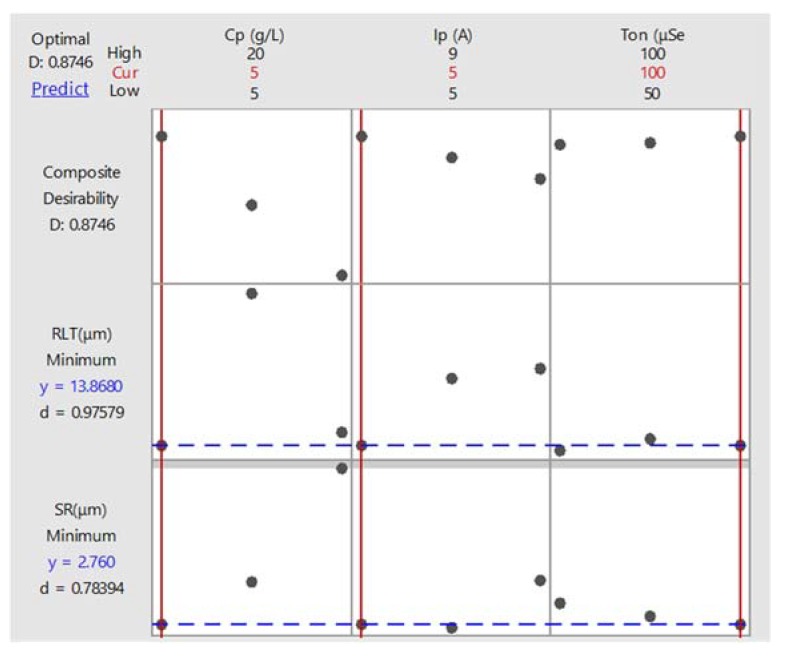
Parametric optimization for minimizing SR and RLT.

Figure 10 shows the optimized results and parameters with the goal to minimize both of the responses. The desirability index comes out to be 87.46%. Table 8 and Table 9 show the optimized parameters and corresponding optimal values of responses, respectively. Overall, this study evaluates surface integrity, which plays an important role in biomedical applications.

## 4. Conclusions

Electric discharge machining of titanium alloy ELI grade 23 is carried out by Si powder mixing in the dielectric. Surface integrity of Ti6Al4V ELI grade 23 was investigated in the present study. The effect of powder concentration (5 g/L, 10 g/L and 20 g/L) along with pulse current and pulse on-time is investigated on micro and nanoscale surface topography. Based on results and discussions, the analysis of scanning electron microscopy, and other statistical analysis, the following conclusions have been inferred:

- The machined surface produced by Si powder (all concentrations) mixed EDM exhibited better surface morphology in terms of reduced degree of surface irregularities (craters, cracks etc.) as compared to conventional (0 g/L) EDM of Ti Alloy.
- Powder concentration (Cp) were found to be most significant factor in Silicon mixed EDM to control surface roughness and recast layer thickness. The percentage contribution of Cp in controlling surface roughness and recast layer thickness is 61.82% and 37.03% respectively.
- RLT is significantly influenced by powder concentration of Si powder, at 5 g/L RLT obtained ~13 μm while increase in concentration up to 20g/L resulted ~34 μm.
- Superior surface characteristics are obtained on the optimized parameters: Si powder (concentration 5 g/L), 5 A peak current and 100:100 µs pulse on:off duration, majorly because of powder’s polishing action resulting crack free surface. Moreover, a nano-porous recast layer with pore size ~50–200 nm has been fabricated on the modified surface.
- EDS analysis confirmed the idea of material migration (possibly composing Ti oxide, Ti carbide, Si oxide, and Si carbide like phases) on the modified surface generating highly favourable chemistry to enhance bio-compatible attributes.

In this research work, surface characteristics of EDM and Si mixed EDM are investigated in the context of biomedical applications. However, standard powder mesh size was used to study surface characteristics through EDM. In future, the effect of different powder mesh sizes can be evaluated on machining efficiency (material removal) and surface characteristics (roughness, crater depth) for different applications. 

## Figures and Tables

**Table 1:** Elemental composition of Ti6Al4V ELI (Grade 23).

Element	Weight %
Al	5.860
V	3.940
Fe	0.134
O	0.100
C	0.017
N	0.008
H	0.00198
Ti	Balance

**Table 2:** Salient properties of workpiece.

Property	Value	Unit
Hardness	320	Vicker D
Density	4.43	g/cm^3^
Yield strength	955	MPa
Ultimate tensile strength	990	MPa
Modulus of elasticity	114	GPa
Thermal conductivity	6.7	W/m-K
Electrical resistivity	178	μΩ-cm
Melting point	1660	°C

**Table 3:** Properties of Si Powder [45].

Property	Value	Unit
Mesh size	>30	μm
Thermal conductivity coefficient @ 293 K	149	W/m-K
Electrical Resistivity at 293 K	3–4	μΩ-cm
Shape of particle	Spherical	-
Color	Black	-

**Table 4:** List of variable control parameters and their values.

Factor Name	Symbol	Unit	Levels		
			−1	0	+1
Powder Concentration	C_P_	g/L	5	10	20
Pulse Current	I_P_	A	5	7	9
Pulse Duration	T_ON:OFF_	μSec	50:100	75:100	100:100

**Table 5:** List of constant parameters and their values.

Parameter	Value
Auxiliary voltage (V)	Middle (150–220)
Spark voltage (V)	5
Arc sensitivity	3
Servo sensitivity	4
Spark time (sec)	5
Flushing time (sec)	5
Dielectric	Kerosene oil

**Table 6:** Analysis of variance of surface roughness.

Source	DF	Adj SS	Adj MS	F Value	P Value	-	PCR %
A: Powder Concentration (g/L)	3	53.408	17.803	51.38	0.000 *	Significant	61.82
B: Pulse Current (A)	2	2.0470	1.0235	2.95	0.091	In-significant	2.369
C: Pulse on Time (μSec)	2	1.7512	0.8756	2.53	0.121	In-significant	2.027
A ∗ B	6	16.089	2.6815	7.74	0.001 *	Significant	18.62
A ∗ C	6	8.5888	1.4315	4.13	0.018 *	Significant	9.941
B ∗ C	4	0.3500	0.0875	0.25	0.903	In-significant	0.405
Error	12	4.1575	0.3465				4.812
Total	35	86.392					100.0

*: Significant at 95% confidence leve.

**Table 7:** Analysis of variance of recast layer thickness.

Source	DF	Adj SS	Adj MS	F Value	P Value		PCR %
A: Powder Concentration (g/L)	2	1055.83	527.91	5.59	0.030 *	Significant	37.03
B: Pulse Current (A)	2	398.20	199.10	2.11	0.184	In-significant	13.96
C: Pulse on Time (μSec)	2	75.67	37.83	0.40	0.683	-	2.654
A ∗ B	4	214.79	53.70	0.57	0.693	-	7.533
A ∗ C	4	246.82	61.71	0.65	0.641	-	8.657
B ∗ C	4	103.69	25.92	0.27	0.887	-	3.637
Error	8	755.96	94.50				26.51
Total	26	2850.97					100.0

*: Significant at 95% confidence level.

**Table 8:** Optimized parameters.

Sr. No.	Input EDM Parameters	Notation	Optimal Settings for SR and RLT		
			Optimal Levels	Level Value	Units
1	Powder Concentration	C_P_	−1	5	g/L
2	Pulse Current	I_P_	−1	5	A
3	Pulse Duration	T_ON:OFF_	+1	100:100	μSec

**Table 9:** Parametric optimization for minimizing SR and RLT.

**Optimal settings**	**Levels of Parameters**	**SR Desirability**	**RLT Desirability**	**Composite Desirability**
	Cp −1, Ip −1, Ton +1	0.7839	0.9757	-
		SR Exp. Value	RLT Exp. Value	0.8746
		2.760	13.868	-
